# The Impact of Stress on Cognitive Performance in Remote Smartphone‐Based Assessments: Exploring Within‐Person Variability

**DOI:** 10.1002/alz70857_102501

**Published:** 2025-12-25

**Authors:** Sarah Prieto, Molly Split, Alyssa N. De Vito, Jennifer Strenger, Nelson A. Roque, Martin J. Sliwinski, Louisa I. Thompson, Zachary J. Kunicki

**Affiliations:** ^1^ Alpert Medical School of Brown University, Providence, RI, USA; ^2^ Butler Hospital Memory Aging Program, Providence, RI, USA; ^3^ Butler Hospital Memory and Aging Program, Providence, RI, USA; ^4^ Brown University Medical School, Providence, RI, USA; ^5^ Bryn Mawr College, Bryn Mawr, PA, USA; ^6^ The Pennsylvania State University, University Park, PA, USA; ^7^ Pennsylvania State University, State College, PA, USA

## Abstract

**Background:**

Although there is an association between stress and cognitive dysfunction in older adults, this research typically uses between‐group comparisons. Advances in remote, smartphone‐based cognitive assessments allow for examination of within‐person variability, including testing how fluctuations in an individual's stress levels influence their own cognitive performance. The current study explored within‐person variability in stress as predictors of cognitive outcomes and examined how these associations differ by amyloid (Aβ) status.

**Method:**

Participants included 123 cognitively normal adults (60‐81 years old, 66.7% female, 86.2% White, mean education = 16.49 years). A subsample had Aβ PET imaging results available (Aβ+ = 25; Aβ− = 48). Participants rated their stress using a single‐item immediately before completing cognitive assessments on the Mobile Monitoring of Cognitive Change (M2C2) smartphone‐based app. Data were gathered three times daily over eight days. Cognitive tasks included processing speed (PS), visual working memory accuracy (WM), and episodic memory accuracy (EM). Multilevel modeling examined the interaction between within‐person stress and session number on cognitive performance, adjusting for between‐person variability in stress, age, sex, and education. Exploratory analyses stratified by Aβ status (Aβ+ vs. Aβ−).

**Result:**

An interaction between within‐person stress and session number emerged for PS (p = .01), such that lower stress ratings were associated with quicker task completion over time. For WM, an interaction with session number was observed (p = .04), suggesting that the positive association between performance and session number was more pronounced when participants reported higher stress levels. No significant interactions emerged when examining EM (p = .23). Exploratory analyses revealed that among Aβ+ individuals, an interaction between within‐person stress and session number for WM (p = .02). No other significant associations were found by Aβ status.

**Conclusion:**

This study highlights the role of stress fluctuations on cognitive performance during remote assessments. Increased stress was linked to slower PS over time, while increased stress was associated with improved WM, particularly among Aβ+ individuals. While chronic stress is typically associated with cognitive dysfunction, intraindividual fluctuations in stress may enhance performance by increasing attentional resources, at least in certain cognitive domains.

